# Supplementary material for: Factors influencing the bias between blood gas analysis versus central laboratory hemoglobin testing. A secondary analysis of a randomized controlled trial
Source: PLoS One. 2020 Oct 30;15(10):e0240721. doi: 10.1371/journal.pone.0240721 (PMC7598475; doi:10.1371/journal.pone.0240721)
Supplement: Attachment — Submitted filename: Rebuttal Letter_Tanner et al_2020.08.16 .docx [file pone.0240721.s001.docx]

PONE-D-20-13621
Factors Influencing the Bias between Blood Gas Analysis versus Central Laboratory Hemoglobin Testing. A secondary analysis of a randomized controlled trial.
PLOS ONE


Journal Requirements:

When submitting your revision, we need you to address these additional requirements.

 1. Please ensure that your manuscript meets PLOS ONE's style requirements, including those for file naming.

>>>Our manuscript was formatted and named according to PLOS ONE style requirement.

2. We note that you have indicated that data from this study are available upon request. PLOS only allows data to be available upon request if there are legal or ethical restrictions on sharing data publicly. For information on unacceptable data access restrictions, please see <http://journals.plos.org/plosone/s/data-availability#loc-unacceptable-data-access-restrictions>.

>>>We added a statement in the attached Cover letter.

Reviewers' comments:

Reviewer's Responses to Questions

**Comments to the Author**

Reviewer #1: • In the introduction, the authors stated: “hemoglobin (Hb) and/or hematocrit levels are one key factor to determine the need for RBC transfusion”. However, many studies have already demonstrated that hemoglobin concentration is not a reliable indicator of transfusion requirement. The authors should add a comment about that.

>>>We appreciate the Reviewer’s insightful suggestion. Indeed, hemoglobin concentration is not an exclusive indicator for transfusion requirement since fluid shifts from massive bleeding, blood transfusion and crystalloid infusion influence hemoglobin concentration. Therefore, guidelines recommend that the decision to transfuse should not be based exclusively on hemoglobin level only and that clinicians should also consider physiological constitution, haemodynamic and spirometry, volume status, and dynamic of bleeding. We included the following statement into the introduction:

“In these critical situations hemoglobin (Hb) and/or hematocrit levels are one key factor *among others* to determine the need for RBC transfusion. *It is noteworthy to mention that transfusion guidelines also recommend to consider physiological constitution, hemodynamic and spirometry, volume status, and dynamic of bleeding.”*

• While in the abstract the objective of the study is “to investigate the accuracy of BGA hemoglobin testing as compared to CL services”, in the main manuscript the declared aim is different, particularly “to elucidate factors influencing the bias between the most common used methods -CL and BGA- to measure Hb levels in surgical patients.” Which one is the right aim?

>>>We agree with the concern raised by the Reviewer and apologize for the resulting confusion. Our aim was to investigate the accuracy of BGA hemoglobin testing compared to central laboratory services. Previous studies demonstrated that measurements differ between BGA and central laboratory. In order to further investigate and quantitate this deviation, we explored several factors that potentially influence parameters measured with BGA. We revised the passages accordingly:

“Here, we performed a sub-analysis of the ongoing “liberal transfusion strategy to prevent mortality and anemia-associated ischemic events in elderly non-cardiac surgical patients” (LIBERAL-Trial) [16] to *investigate the accuracy of BGA Hb testing compared to CL by determining factors that potentially influence Hb measurements in surgical patients.*“

“Here, *we investigated the accuracy of BGA compared to CL by determining factors that can potentially influence these measurements.*”

• In the study design section, the authors stated that elderly patients scheduled for surgery were included in the trial, but they do not clarify the lower limit of age for inclusion in the study.

>>>We thank the Reviewer for this notification and included the missing information:

“Briefly, elderly patients *(≥70 years)* scheduled for *intermediate or high risk non-cardiac* surgery were included in the trial and randomized to a restrictive or liberal transfusion group as soon as Hb level dropped below ≤ 9 g/dl during surgery or postoperative day 1 to 3.”

• In the result section, the authors stated: “mean bias was reduced by
0.02 g/dl in patients after 24 hours of volume substitution (-0.37 ± 0.29 g/dl) compared to values immediately after surgery (-0.39 ± 0.26 g/dl)”. However, p value is not statistically significant (p=0.202).

>>>We thank the Reviewer for this important comment and included “non-significantly” into the sentence:

“Mean bias was *non-significantly* reduced by 0.02 g/dl in patients after 24 hours of volume substitution (-0.37 ± 0.29 g/dl) compared to values immediately after surgery (-0.39 ± 0.26 g/dl) (p=0.202) (Table 1).”

• The authors did not discuss the impact of their results on the clinical practice. How could a physician use these results in his daily work when deciding whether to transfuse a patient?

>>>We thank the Reviewer for this suggestion and included the following passage into the discussion section:

“*Our analysis confirms that BGA produces stable values for Hb and is not influenced by the patient’s physiology except for smoker. This observation is especially relevant during trauma setting where no information about the critical patient is available. The bias of smoker is only minor and therefore, we believe, negligible in this setting. Hemoglobin measurement is not only important for categorizing the patients’ trauma but is also used in several scoring systems, such as the Trauma Associated Severe Hemorrhage Score (TASH) [30]. Prior to RBC transfusion, the physician evaluates if there are possible transfusion triggers such as low Hb values, hypotension, tachycardia or lactatemia. In these settings, measurements* particularly in intensive care units where Hb is frequently determined [22, 23], *with BGA are recommended since less blood volume is required, turn-around time is faster, and stable results for Hb can be observed.*”

Reviewer #2: The authors set out to study Bias between CL and three different BGA machines. One of them was Co-oximeter. Ideally the study design for this research question would be significantly different from what the authors have used. The design would involve a single blood sample taken per patient and sent for CL and BGA analysis, with stringent control of analytical factors related to specimen collection, transport and processing.

>>>We appreciate the Reviewers raised concern.

The ongoing LIBERAL trial with a target sample size of more than 2,400 surgical patients, currently comprising >1,800 pairs of Hb measurements, allowed us to investigate the accuracy of BGA Hb testing compared to CL. As outlined in our limitation section, we unfortunately were not able to compare pairs of one blood sample. We agree, that a single blood sample used for both measurements would be preferred, however in this setting clinical routine would be affected since involvement of additional trained clinical staff is necessary. Furthermore, additional ethical approval would be required in order to obtain extra blood samples that are not included in the study protocol of the LIBERAL trial.

The range of Hb, which is of clinical interest – i.e. where the decisions regarding transfusion are usually made (6 to 8 gm/dl), is poorly represented. Ideally the ranges of Hb should be equally represented <6, 6-8, >8…for example.

We thank the Reviewer for this interesting perspective and included additional information into the results section:

*“Overall, 1,389 Hb pairs were measured with Hb ≥8 g/dl and 184 Hb pairs between 6 and 8 g/dl.”*

Additionally, we included a sentence about the unequal representation of Hb pairs within the target range into the limitation section:

*“Ideally, equal number of Hb pairs for the different ranges of Hb ≤6, 6-8, and ≥8 g/dl would be preferred for analysis. However, our observed distribution of higher amount of Hb pairs for 8 g/dl which are followed by 6-8 g/dl represent the clinical distribution of Hb values of patients undergoing major surgical procedures.”*

I don’t agree with Authors claiming that the bias decreases with lower Hb values. Firstly, there is no explanation provided that why this might be true and secondly, as mentioned before the low range is poorly represented.

We thank the Reviewer for this critical comment. In order to investigate whether the bias decreases with lower Hb levels, we displayed the bias in Hb compared to CL in percentage.

*“The LOWESS curve for absolute Hb values showed that low Hb values are associated with smaller bias. Hb values of 10 to 15 g/dl display a bias with -0,5 g/dl whereas Hb values of 6 g/dl display a bias of -0,25 g/dl. (Figure 3). However, Hb differences plotted as percentage revealed an increase of up to 5% of the Hb value that are associated with smaller values (Figure 4).”*

*“Figure 4: Bland-Altman Plot and LOWESS curve showing differences in percentage of Hb pairs (LIB-07 and LIB-26).*

*The difference in percentage of the Hb values (Hb-CL and Hb-BGA) calculated for each pair and plotted against the mean value of both measurements are displayed ((Hb-CL + Hb-BGA)/2). The LOWESS curve (red line) shows the tendency of mean bias expressed as percentage of CL Hb with lower Hb levels. Hb= hemoglobin, CL = central lab, BGA = blood gas analysis”*

We really thank the Reviewer for the raised concern and incorporated the new results as Figure 4 into the result section. Overall, we agree with the reviewer that the bias increases relatively with lower Hb values. As suggested by the reviewer, we cannot make a statement for Hb pairs lower than 6 g/dl.

We therefore added this statement to our discussion:

*“Our analysis showed that bias increases relatively with lower Hb values. However, clinically these findings are minimal (0.25 g/dl for Hb pairs from 6 to 8 g/dl). This Hb range is used for transfusion decision and we expect no unnecessary or delayed RBC transfusion, and no major impact on the LIBERAL-Trial performance.”*

The authors then go on to analyse the factors that impact the bias. They have included several factors (patient related and treatment related), without explanation or rationale as to why they included these factors. This exercise then appears to be similar to ‘data mining’ exercise rather than carefully thought of hypothesis generation and testing.

>>>We thank the Reviewer for this critical remark. Measurement values differ between BGA and central laboratory services. For example, Giraud et al compared the accuracy of four different bedside devices with CL. The analysis of 219 measurements from 53 patients revealed for each device different Hb levels. Among all methods, the point-of-care testing (POCT) device HemoCue® displayed the smallest and CO-Oximetry the biggest bias [Giraud et al Br J Anaesth, 2013]. Additionally, studies investigated whether differences in clinical routines or patient’s physiological status affect Hb values using different devices for measurements. Until now, the bias between BGA measurement and CL depending on the patient’s physiology, co-medication and clinical events such as blood transfusion or intraoperative volume therapy has not yet been assessed. Several studies compared surgical outcome in patients receiving either a restrictive or liberal transfusion regime. In these studies, different methods have been used in different heterogenous patient populations to detect Hb concentration, however details about the used method for measurement and impact of patient characteristics are often not described. In addition, it is not evident whether the same methods were used in multicenter studies or more importantly considered during the analysis [Villanueva et al Nat Rev Gastroenterol Hepatol, 2015, Carson et al N Engl J Med, 2011, Mazer et al N Engl J Med, 2017, Holst et al N Engl J Med, 2014.]. Since accurate measurement of Hb level is essential within the LIBERAL trial we sought to investigate the accuracy of BGA used in our trial compared to CL regarding Hb measurement and to determine factors that potentially influence these measurements. The selection of analysed factors was based on published results [Carson et al N Engl J Med, 2011, Mazer et al N Engl J Med, 2017, Holst et al N Engl J Med, 2014], discussed clinical relevance within the publications and collected data from our study protocol (Meybohm, P., et al., Liberal transfusion strategy to prevent mortality and anaemia-associated, ischaemic events in elderly non-cardiac surgical patients - the study design of the LIBERAL-Trial. Trials, 2019), but was not associated with data mining.

Authors have not taken into consideration the fact that one patient is represented in the data more than once. This fact is important when analysing patient related factors. This is also important as the three centres were very different. For example the Lib07 centre had 6 times more specimens than the other centres. However, the number of patients of the Lib07 and Lib26 combined were only 1/3rd of the entire sample size (result section 2nd Para). This means the number of samples per patient were much more in Lib 07 than others.

We absolutely agree and highly appreciate this Reviewer’s insightful comment. Multiple Hb measurements per patient provided unequal numbers of replicated data in pairs. To adjust for multiple testing a linear mixed-effect model could be applied, which, however, is associated with less robust results. We therefore used robust statistical methods such as the median and median absolute deviation to investigate the accuracy of BGA compared to CL.

I am concerned that the authors have found incidental findings in a poor study design, which are further poorly substantiated in the discussion section.

>>>We acknowledge the concern raised by the Reviewer. The LIBERAL trial is a multicentred, controlled clinical trial including 2470 elderly patients undergoing intermediate to high risk surgery. Previous high ranked trials investigated Hb trigger for different clinical situations such as hip surgery, cardiac surgery or septic shock (Carson et al N Engl J Med, 2011, Mazer et al N Engl J Med, 2017, Holst et al N Engl J Med, 2014). The LIBERAL Trial, however, explores the optimal Hb trigger for elderly anemic patients who undergo surgery since they form a great part of the current patient collective in hospitals and the optimal transfusion threshold has not been investigated yet. This protocol enabled us to analyse a large number of different patients including their Hb values, physiology, pre-existing conditions and medications in context of clinical events such as bleeding, anemia and blood transfusion. Ultimately, our trial-patients represent a great patient collective and therefore ensure high clinical relevance. The study protocol has recently been published ((Meybohm et al. 2019)

In addition, we hope that our revised version of manuscript including suggestions from three additional reviewers improved our manuscript and presentation of our data.

The Authors do talk about smokers and MetHb. The Cooximeter should be able to pick this up in contrast to other BGL machines assessed. So did this effect the bias in the Lib05 centre?

We thank the Reviewer for this suggestion. In order to avoid interference with different standard deviations from the trial sites that may be caused by the different analytic devices, we focused on LIB-07 and LIB-26. However, as suggested by the Reviewer we analysed the smoker effect for LIB-05 and found a significantly reduced bias for this centre, too. If the reviewer would agree, these results cannot be regarded as stable since only 14 values of 5 patients could be analysed.

The Authors have used regression analysis to study several factors. Apart from above criticism of lack of rationale/procedure in selecting factors, there is a lot unknown regarding collinearity of factors, excessive representation of one patient etc. Was the assumption of normality met?

We thank the reviewer for this critical remark. Generally, we used robust statistical methods, such as the median and median absolute deviation which are tolerant against outliners. For these robust statistical methods, no normality was required. Overall, our analysis for factors influencing the bias in Hb measurement is explorative. The aim was to elucidate whether Hb measurement is influenced by these factors. Furthermore, outliners were excluded if bias deviated by five times standard deviation from the median. We adjusted the method section accordingly:

*“Outliners were excluded if bias displayed a value 5 times of standard deviation of the respective median value.”*

The authors have discussed about bias, but the biases appear to be minimal which makes you wonder if its all clinically meaningful. Also authors have used statements such as “Mean bias was reduced by 0.02 g/dl in pts after 24 hours of volume substitution compared to values after surgery” (In results section). Basically there is no difference given the P value and insignificant difference. Hence commenting as the bias is reduced, is incorrect.

>>>We thank the Reviewer for this perspective. The LIBERAL Trial is a prospective, randomized, multicentre, controlled clinical phase IV trial randomising (≥ 70 years) patients undergoing intermediate- or high-risk non-cardiac surgery. Registered patients will be randomised only if Hemoglobin reaches ≤9 g/dl during surgery or within 3 days after surgery either to the LIBERAL group (transfusion of a single RBC unit when Hb ≤ 9 g/dl with a target range for the post-transfusion Hb level of 9–10.5 g/dl) or the RESTRICTIVE group (transfusion of a single RBC unit when Hb ≤ 7.5 g/dl with a target range for the post-transfusion Hb level of 7.5–9 g/dl). Since it is known that measurement values differ between BGA and Central Laboratory services, we investigated the accuracy of BGA Hb testing compared to CL in participating sites. Our aim was to elucidate whether a bias exists and should be considered within the ongoing LIBERAL trial.

According to reviewer suggestion we included “non-significantly” into the sentence:

“Mean bias was *non-significantly* reduced by 0.02 g/dl in patients after 24 hours of volume substitution (-0.37 ± 0.29 g/dl) compared to values immediately after surgery (-0.39 ± 0.26 g/dl) (p=0.202) (Table 1).”

To further acknowledge the reviewer comments we further added to the manuscript:

*“Our analysis confirms that BGA produces stable values for Hb and is not influenced by the patient’s physiology except for smoker. This observation is especially relevant during trauma setting where no information about the critical patient is available. The bias of smoker is only minor and therefore, we believe, negligible in this setting. Hemoglobin measurement is not only important for categorizing the patients’ trauma but is also used in several scoring systems, such as the Trauma Associated Severe Hemorrhage Score (TASH) [30]. Prior to RBC transfusion, the physician evaluates if there are possible transfusion triggers such as low Hb values, hypotension, tachycardia or lactatemia. In these settings, measurements* particularly in intensive care units where Hb is frequently determined [22, 23], *with BGA is recommended since less blood volume is required, turn-around time is faster, and stable results for Hb can be observed.”*

The authors took “results within two hours” to define a pair. The actual time of sampling could be 20 minutes to 60 minutes prior to results.
How did they figure out that there was an intervention between the sampling for BGA and CL? How did they manage these pairs where there was an intervention?

We thank the Reviewer for the raised point. Hb pairs were considered as outliners if bias had a value 5 times of standard deviation of median value and were excluded from analysis (Figure 3 and 4). However, we cannot fully exclude that there was no intervention between Hb pairs.

*“Outliners were excluded if bias displayed a value 5 times of standard deviation of the respective median value.”*

We cant compare venous and arterial blood. Authors do acknowledge that in their limitations. I am surprised by their use of word “might”. It definitely affects the bias. We don’t know what was the extent of this confounding factor.

>>>We thank the reviewer for this comment and adjusted our limitation section accordingly:

“Finally, *due to the study protocol* we *were not able to* differentiate between arterial or venous samplings which *may* also affect the bias due to possible differences in plasma volume. *As BGA analysis is frequently used in clinical practice to assess Hb status and potentially trigger clinical decisions, we suggest that future trials should consistently use either venous or arterial blood for analysis.*”

Reviewer #3: In this manuscript, dr Meybohm and colleagues present results of a secondary analysis of an ongoing mRCT on management of blood transfusion after surgery. The aim of their study is to compare Hb levels measured with BGA to Hb levels obtained through central laboratory analysis. They identified several factors that could potentially bias the results of HB analysis by BGA versus CL analysis.

As BGA analysis is frequently used in clinical practice to assess Hb status and potentially trigger clinical decisions, the results of this study may be potentially relevant for several clinicians.

I have a few comments for the Authors which I hope will help them to improve their manuscript:
1. In my opinion, the Authors should better detail in the Discussion the clinical relevance of their findings, and how the results of their study may affect clinical practice and the ongoing LIBERAl trial. E.g. is a bias of 0.09 g/dL clinically relevant?

>>>We highly appreciate all suggestions made by the reviewer and added the following sentences into the discussion:

*“A small bias is negligible and clinicians should not hesitate to trust measurements using BGA devices.* Nevertheless, we suggest that hospitals assess their individual bias before implementing BGA as valid and stable supplement to CL.”

AND

*“Our analysis shows that BGA is a stable supplement to CL to measure Hb values. In this sub-analysis of the LIBERAL trial, we found that Hb values measured by BGA vary between different centers. To allow a precise analysis each participating centre should determine their individual correction factor which will be applied during our final analysis.* This study illustrates the importance of determining the concordance between values obtained by BGA and those obtained in the CL for each individual hospital.”

AND

“*Our analysis confirms that BGA produces stable values for Hb and is not influenced by the patient’s physiology except for smoker. This observation is especially relevant during trauma setting where no information about the critical patient is available. The bias of smoker is only minor and therefore, we believe, negligible in this setting. Hemoglobin measurement is not only important for categorizing the patients’ trauma but is also used in several scoring systems, such as the Trauma Associated Severe Hemorrhage Score (TASH) [30]. Prior to RBC transfusion, the physician evaluates if there are possible transfusion triggers such as low Hb values, hypotension, tachycardia or lactatemia. In these settings, measurements* particularly in intensive care units where Hb is frequently determined [22, 23], *with BGA is recommended since less blood volume is required, turn-around time is faster, and stable results for Hb can be observed.*”

2. The Authors should better underline in the introduction the difference between their work and previously published studies on difference between BGA and CL analysis. What does this study add to current knowlegde?

>>>We thank the reviewer for this important remark and revised our manuscript accordingly:

“Several methods, such as central laboratory (CL), blood gas analysis (BGA) or CO-Oximetry [11] are applied to evaluate the Hb level, of which BGA and CL are the most common used methods intraoperatively. However, measurement values differ between BGA and CL [4-7]. *Studies compared different devices in order to establish the optimal, less invasive, fastest but at the same time most accurate method to measure Hb level. For example,* Giraud et al compared the accuracy of four different bedside devices with CL. The analysis of 219 measurements from 53 patients revealed for each device different Hb levels. Among all methods, the point-of-care testing (POCT) device HemoCue® displayed the smallest and CO-Oximetry the biggest bias [3]. Non-invasive Hb measurement, such as spectrophotometry, turned out to be inferior to invasive Hb measurement (BGA) [8]. *Additionally, studies investigated whether differences in clinical routines or patient’s physiological status affect Hb values using different devices for measurements*. For example, the concentration of Hb varies depending on the amount of plasma volume, leading to different Hb levels between arterial or venous samples within the same patient. In addition, position of the body or time of the day during blood withdrawal also appear to influence Hb level [9]. In cases of hemodilution with hematocrit below 30%, measurement of Hb concentration using CO-Oximetry seems to be more accurate than conductivity [10]. Ng and colleagues evaluated the bias in Hb level between CL and POCT device before and after major blood loss (≥ 25% of blood volume). The analysis revealed that Hb levels were lower when measured with POCT device as compared to laboratory. Therefore, the authors recommend physicians not to use the POCT device in particular situation such as extensive blood loss when accurate measurement is essential [11]. *However, until now, the bias between BGA measurement and CL depending on the patient’s physiology, co-medication and clinical events such as blood transfusion or intraoperative volume therapy has not been assessed.* Additionally, accurate measurement of Hb level is important for studies ultimately implementing clinical guidelines. Several studies compared surgical outcome in patients receiving either a restrictive or liberal transfusion regime. In these studies, several methods have been used to detect Hb concentration, however details about the used method for measurement are often not described. In addition, it is not evident whether the same methods were used in multicenter studies or more importantly considered during the analysis [12-15].

3. I understand that blood samples for BGA and CL analysis were taken at different time-points. However, this is not entirely clear from the Methods. I suggest to describe the blood sampling timing in a specific paragraph "blood sampling"

>>>We incorporated the comment made by the Reviewer by including a new section within the methods:

*“Blood Sampling*

*During hospital stay, Hb measurement by CL or BGA occurred regularly. Pairs of Hb were taken in the postanaesthesia care unit after surgery. Furthermore, if the patient remained for 24 hours in the postanaesthesia care unit, another pair was taken the following day. If patients were admitted to the intensive care unit or intermediate care unit, pairs of Hb were taken at least twice a day. During massive bleeding or before RBC transfusion, additional blood samples were analysed using CL or BGA.”*

4. Do the Authors have data on BGA and CL agreement during massive transfusion/blood loss? This would be a interesting subgroup analysis

>>> As pointed out by the reviewer, a subgroup analysis to assess the bias during massive bleeding would be very interesting. Ng and colleagues evaluated the bias in Hb level between CL and POCT device before and after major blood loss (≥ 25% of blood volume). The analysis revealed that Hb levels were lower when measured with POCT device as compared to laboratory. Therefore, the authors recommend physicians not to use the POCT device in particular situation such as extensive blood loss when accurate measurement is essential (Ng et al. 2014). We agree that further studies are needed to confirm this finding. We defined massive bleeding as “massive blood loss” listed as indication for RBC transfusion in our study report file, however, only six patients qualified for this event. In addition, no pairs of Hb were taken during massive bleeding since BGA testing was mainly used and less CL measurements. From our point of view, these data are not representative and were not included in our analysis.

5. The Authors frequently refer to "volume therapy". However, it is unclear what does this mean and how was assessed. Do the Authors refer to maintenance fluids, volume resuscitation, fluid balance? How were these assessed/managed?

>>>We have acknowledged this comment by adding the following information into the method section.

*“Volume therapy refers to the administration of crystalloid fluids during surgery. Often, 500mL to 1L crystalloid solutions are used. Immediately before surgery, crystalloids are applied to resuscitate the deficit in volume from fasting. During the surgery, crystalloid solutions are applied in order to resuscitate the deficit in blood volume from perspiration or bleeding by using physiological marker such as lactate, blood pressure and heart rate. Blood loss and the amount of crystalloids were not measured. Because volume therapy is part of our perioperative guidelines, we compared Hb pairs after volume therapy with postoperative Hb pairs.”*

6. Please move the list of medications included in the analysis in the Methods section rather than in the Results section

>>>We thank the reviewer for this comment and moved the list of medications from the result into the method section.

7. The sentence "The LOWESS curve showed a tendency of the bias with decreasing Hb levels." is unclear, please edit.

>>>We apologize and adjusted the sentence accordingly:

*“The LOWESS curve for absolute Hb values showed that low Hb values are associated with smaller differences. Hb values of 10 to 15 g/dl display a bias with -0,5 g/dl whereas Hb values of 6 g/dl display a bias of -0,25 g/dl.”*

8. Do the Authors have data on bias of BGA vs CL assessed before, during, and after surgery?

>>>We absolutely agree with this interesting aspect. Within the LIBERAL-trial only one Hb value, either CL or BGA, was taken as baseline and therefore no comparison was feasible. During surgery, Hb was mainly measured by BGA. We included the following sentence into our limitation section:

*“Furthermore, we did not analyse the bias of pre- or intraoperative Hb pairs.*

Reviewer #4: This is an interesting subanalysis of the LIBERAL trial, and the restriction to centres with a large number of cases is a sensible one here in the first instance - one does of course want to know whether case volume actually affects accuracy and this could be usefully explored.

What we have here is a number of paired data points by 2 different methods, but also >1 point per patient. So there is clustering here as well and the pairs are not independent. The statistical methodology needs to allow for this but nowhere is this multiplicity of measurements and how it is handled described. This is particularly important in the case of the predictors of bias.

We sincerely appreciate the Reviewer’s insightful comment.

Multiple Hb measurements per patient provided unequal numbers of replicated data in pairs. To adjust for multiple testing a linear mixed-effect model could be applied, which, however, is associated with less robust results. We therefore used robust statistical methods such as the median and median absolute deviation to investigate the accuracy of BGA compared to CL.

>>> We added following sentence to our method section:

*“Because we expected no differences to our results, we did not adjust for multiplicity.”*

Furthermore, we excluded outliners if bias displayed a value 5 times of standard deviation of the respective median value, for example in cases of RBC transfusion or massive blood loss during the 2h time range.

In Figure 3 it would be interesting to look at percentage bias as well here just to see if the trend here actually represents a different type of measurement error. The issue of whether variance depends on value is also interesting and could usefully be explored.

>>>We agree with the Reviewer and have incorporated this suggestion by displaying the bias in Hb compared to CL in percentage. We included a new Figure (Figure 4) and revised the result section accordingly:

*“The LOWESS curve for absolute Hb values showed that low Hb values are associated with smaller bias. Hb values of 10 to 15 g/dl display a bias with -0,5 g/dl whereas Hb values of 6 g/dl display a bias of -0,25 g/dl. (Figure 3). However, Hb differences plotted as percentage revealed an increase of up to 5% of the Hb value that are associated with smaller values (Figure 4).”*

Furthermore, we assessed whether ranges of Hb levels is equally represented and included additional information into the results section:

*“Overall, 1,389 Hb pairs were measured with Hb ≥8 g/dl and 184 Hb pairs between 6 and 8 g/dl.”*

Additionally, we included a sentence about the unequal representation of Hb pairs within the target range into the limitation section:

*“Ideally, equal number of Hb pairs for the different ranges of Hb ≤6, 6-8, and ≥8 g/dl would be preferred for analysis. However, our observed distribution of higher amount of Hb pairs for 8 g/dl which are followed by 6-8 g/dl represent the clinical distribution of Hb values of patients undergoing major surgical procedures.”*
